# West Nile Virus and Other Nationally Notifiable Arboviral Diseases — United States, 2015

**DOI:** 10.15585/mmwr.mm6602a3

**Published:** 2017-01-20

**Authors:** Elisabeth Krow-Lucal, Nicole P. Lindsey, Jennifer Lehman, Marc Fischer, J. Erin Staples

**Affiliations:** ^1^Epidemic Intelligence Service, CDC; ^2^Division of Vector-Borne Diseases, National Center for Emerging and Zoonotic Infectious Diseases, CDC.

Arthropod-borne viruses (arboviruses) are transmitted to humans primarily through the bites of infected mosquitoes and ticks. The leading cause of domestically acquired arboviral disease in the United States is West Nile virus (WNV) (*1*). Other arboviruses, including La Crosse, St. Louis encephalitis, Jamestown Canyon, Powassan, and eastern equine encephalitis viruses, also cause sporadic cases and outbreaks. This report summarizes surveillance data reported to CDC in 2015 for nationally notifiable arboviruses. It excludes dengue, chikungunya, and Zika viruses, which are primarily nondomestic viruses typically acquired through travel (and are addressed in other CDC reports). In 2015, 45 states and the District of Columbia (DC) reported 2,282 cases of domestic arboviral disease. Among these cases, 2,175 (95%) were WNV disease and 1,455 (67%) of those were classified as neuroinvasive disease (meningitis, encephalitis, or acute flaccid paralysis). The national incidence of WNV neuroinvasive disease was 0.45 cases per 100,000 population. Because arboviral diseases continue to cause serious illness, maintaining surveillance is important to direct prevention activities such as reduction of vector populations and screening of blood donors.

Arboviruses are maintained in a transmission cycle between arthropods and vertebrate hosts. Humans primarily become infected when bitten by an infected tick or mosquito. Person-to-person transmission of domestic arboviruses has been reported through blood transfusion and organ transplantation (*3*). Most human infections are asymptomatic; symptomatic infections commonly manifest as a systemic febrile illness, and, less commonly, as neuroinvasive disease.

Most endemic arboviral diseases are nationally notifiable and are reported to CDC through ArboNET, a national arboviral surveillance system managed by CDC and state health departments (*2*,*3*). Using standard definitions, human cases with laboratory evidence of recent arboviral infection are classified as neuroinvasive or nonneuroinvasive disease (*2*). Cases reported as encephalitis, meningitis, or acute flaccid paralysis are collectively referred to as neuroinvasive disease; others are considered nonneuroinvasive disease. Acute flaccid paralysis can occur with or without encephalitis or meningitis. In this report, any case reported as acute flaccid paralysis (with or without another clinical syndrome) was classified as acute flaccid paralysis and not included in the other categories. Because ArboNET is a passive surveillance system, detection and reporting of neuroinvasive disease is thought to be more consistent and more complete than nonneuroinvasive disease. For this reason, incidence rates were calculated using neuroinvasive disease cases and U.S. Census 2015 mid-year population estimates.

During 2015, a total of 2,282 cases of domestic arboviral disease were reported to CDC. Cases were caused by WNV (2,175 cases, 95%), La Crosse virus (55), St. Louis encephalitis (23), Jamestown Canyon virus (11), Powassan virus (seven), eastern equine encephalitis virus (six), unspecific California serogroup virus (four), and Cache Valley virus (one). Of the 3,141 U.S. counties, 611 (19%) reported one or more cases of arboviral disease. No cases of domestic arboviral disease were reported from Alaska, Hawaii, New Hampshire, Rhode Island, or Vermont.

The 2,175 WNV disease cases were reported from 506 counties in 43 states and DC, including 1,455 (67%) that were neuroinvasive, and 1,804 (83%) with illness onset during July–September (Table 1). The median age of patients was 58 years (interquartile range [IQR] = 46–69 years), and 1,289 (59%) patients were male. A total of 1,616 (74%) patients with WNV disease were hospitalized, and 146 (7%) died. The median age of patients who were hospitalized was 61 years (IQR = 50–73 years), and 996 (62%) were male. The median age of patients who died was 76 years (IQR = 66–83 years), and 94 (64%) were male.

**TABLE 1:** Number and percentage of reported cases of West Nile virus and other arboviral diseases, by virus type and selected patient characteristics — United States, 2015*

Characteristic	Virus type					
	West Nile (N = 2,175)	La Crosse (N = 55)	St. Louis encephalitis (N = 23)	Jamestown Canyon (N = 11)	Powassan (N = 7)	Eastern equine encephalitis (N = 6)
	No. (%)	No. (%)	No. (%)	No. (%)	No. (%)	No. (%)
**Age group (yrs)**						
<18	54 (2)	51 (93)	0 (0)	1 (9)	0 (0)	1 (17)
18–59	1,108 (51)	1 (2)	9 (39)	6 (55)	1 (14)	2 (33)
≥60	1,013 (47)	3 (5)	14 (61)	4 (36)	6 (86)	3 (50)
**Sex**						
Male	1,289 (59)	31 (56)	15 (65)	6 (55)	5 (71)	6 (100)
Female	886 (41)	24 (44)	8 (35)	5 (45)	2 (29)	0 (0)
**Period of illness onset**						
January‒March	2 (0)	0 (0)	0 (0)	0 (0)	0 (0)	0 (0)
April‒June	60 (3)	7 (13)	3 (13)	5 (45)	4 (57)	1 (17)
July‒September	1,804 (83)	47 (85)	19 (83)	5 (45)	1 (14)	5 (83)
October‒December	309 (14)	1 (2)	1 (4)	1 (9)	2 (29)	0 (0)
**Clinical syndrome**						
Nonneuroinvasive	720 (33)	4 (7)	4 (17)	5 (45)	1 (14)	0 (0)
Neuroinvasive	1,455 (67)	51 (93)	19 (83)	6 (55)	6 (86)	6 (100)
Encephalitis	753 (35)	40 (73)	12 (52)	4 (36)	6 (86)	2 (33)
Meningitis	637 (29)	10 (18)	7 (30)	1 (9)	0 (0)	2 (33)
Acute flaccid paralysis^†^	118 (5)	2 (4)	1 (4)	1 (9)	0 (0)	0 (0)
Other	20 (1)	0 (0)	1 (4)	1 (9)	1 (14)	0 (0)
**Outcome**						
Hospitalization	1,616 (74)	52 (95)	19 (83)	9 (82)	7 (100)	6 (100)
Death	146 (7)	0 (0)	2 (9)	0 (0)	1 (14)	4 (67)

* Four unspecified California serogroup virus cases and one Cache Valley virus case also were reported.

^†^ Of the 118 West Nile virus disease patients with acute flaccid paralysis, 91 (77%) also had encephalitis or meningitis.

Among the 1,455 WNV neuroinvasive disease cases, 686 (47%) were reported as encephalitis, 613 (42%) as meningitis, 118 (8%) as acute flaccid paralysis, and 20 (1%) as other neurologic signs or symptoms. Among the 118 patients with reported acute flaccid paralysis, 91 (77%) also had encephalitis or meningitis. Among patients with neuroinvasive disease, 1,382 (95%) were hospitalized, and 142 (10%) died. The incidence of neuroinvasive WNV disease in the United States was 0.45 per 100,000 population (Table 2). The states with the highest incidence rates included California (1.49 per 100,000), North Dakota (1.32), South Dakota (1.28), and Oklahoma (1.25) (Table 2) (Figure). Sixty-one percent of all neuroinvasive disease cases were reported from California (585 cases) and Texas (196). The incidence of WNV neuroinvasive disease increased with age, from 0.04 per 100,000 children aged <18 years to 1.36 in adults aged ≥70 years. The incidence was higher among males (0.57 per 100,000) than among females (0.34).

**TABLE 2:** Number and rate* of reported cases of arboviral neuroinvasive disease, by virus type, U.S. Census division, and state — United States, 2015

U.S. Census division/State	Virus type					
	West Nile	La Crosse	St. Louis encephalitis	Jamestown Canyon	Powassan	Eastern equine encephalitis
	No. (Rate)	No. (Rate)	No. (Rate)	No. (Rate)	No. (Rate)	No. (Rate)
United States	1,455 (0.45)	51 (0.02)	19 (0.01)	6 (<0.01)	6 (<0.01)	6 (<0.01)
**New England**	**16 (0.11)**	**—**	**—**	**1 (0.01)**	**4 (0.03)**	**1 (0.01)**
Connecticut	8 (0.22)	—	—	—	—	—
Maine	1 (0.08)	—	—	—	1 (0.08)	1 (0.08)
Massachusetts	7 (0.1)	—	—	1 (0.01)	3 (0.04)	—
New Hampshire	—	—	—	—	—	—
Rhode Island	—	—	—	—	—	—
**Mid Atlantic**	**82 (0.2)**	**—**	**—**	**1 (<0.01)**	**1 (<0.01)**	**3 (0.01)**
Vermont	—	—	—	—	—	—
New Jersey	23 (0.26)	—	—	1 (0.01)	1 (0.01)	—
New York	42 (0.21)	—	—	—	—	3 (0.02)
Pennsylvania	17 (0.13)	—	—	—	—	—
**E. North Central**	**112 (0.24)**	**29 (0.06)**	**—**	**3 (0.01)**	**1 (<0.01)**	**—**
Illinois	51 (0.4)	—	—	—	—	—
Indiana	16 (0.24)	—	—	—	—	—
Michigan	16 (0.16)	—	—	—	—	—
Ohio	23 (0.2)	23 (0.2)	—	1 (0.01)	—	—
Wisconsin	6 (0.1)	6 (0.1)	—	2 (0.03)	1 (0.02)	—
**W. North Central**	**82 (0.39)**	**1 (<0.01)**	**—**	**1 (<0.01)**	**—**	**—**
Iowa	4 (0.13)	—	—	—	—	—
Kansas	12 (0.41)	1 (0.03)	—	—	—	—
Minnesota	3 (0.05)	—	—	1 (0.02)	—	—
Missouri	23 (0.38)	—	—	—	—	—
Nebraska	19 (1.0)	—	—	—	—	—
North Dakota	10 (1.32)	—	—	—	—	—
South Dakota	11 (1.28)	—	—	—	—	—
**S. Atlantic**	**76 (0.12)**	**17 (0.03)**	**—**	**—**	**—**	**1 (<0.01)**
Delaware	—	—	—	—	—	—
District of Columbia	3 (0.45)	—	—	—	—	—
Florida	12 (0.06)	—	—	—	—	—
Georgia	13 (0.13)	2 (0.02)	—	—	—	—
Maryland	31 (0.52)	—	—	—	—	—
North Carolina	4 (0.04)	11 (0.11)	—	—	—	1 (0.01)
South Carolina	—	1 (0.02)	—	—	—	—
Virginia	13 (0.16)	—	—	—	—	—
West Virginia	—	3 (0.16)	—	—	—	—
**E South Central**	**36 (0.19)**	**3 (0.02)**	**—**	**—**	**—**	**—**
Alabama	5 (0.1)	—	—	—	—	—
Kentucky	1 (0.02)	—	—	—	—	—
Mississippi	25 (0.84)	—	—	—	—	—
Tennessee	5 (0.08)	3 (0.05)	—	—	—	—
**W South Central**	302 (0.77)	1 (<0.01)	—	—	—	1 (<0.01)
Arkansas	16 (0.54)	—	—	—	—	—
Louisiana	41 (0.88)	1 (0.02)	—	—	—	1 (0.02)
Oklahoma	49 (1.25)	—	—	—	—	—
Texas	196 (0.71)	—	—	—	—	—
**Mountain**	**156** (**0.66**)	**—**	**19 (0.08)**	**—**	**—**	**—**
Arizona	67 (0.98)	—	19 (0.28)	—	—	—
Colorado	57 (1.04)	—	—	—	—	—
Idaho	5 (0.3)	—	—	—	—	—
Montana	3 (0.29)	—	—	—	—	—
Nevada	4 (0.14)	—	—	—	—	—
New Mexico	12 (0.58)	—	—	—	—	—
Utah	5 (0.17)	—	—	—	—	—
Wyoming	3 (0.51)	—	—	—	—	—
**Pacific**	**593** (**1.13**)	**—**	**—**	**—**	**—**	**—**
Alaska	—	—	—	—	—	—
California	585 (1.49)	—	—	—	—	—
Hawaii	—	—	—	—	—	—
Oregon	—	—	—	—	—	—
Washington	8 (0.11)	—	—	—	—	—

* Per 100,000 population, based on July 1, 2015, U.S. Census population estimates.

**FIGURE:**
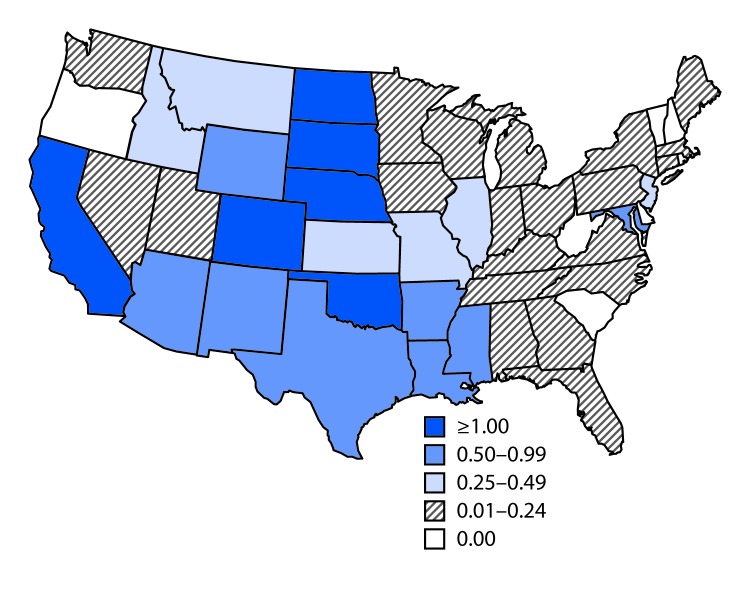
Rate* of reported cases of West Nile virus neuroinvasive disease — United States, 2015 * Per 100,000 population.

Fifty-five La Crosse virus disease cases were reported from 10 states. Of these, 51 (93%) were neuroinvasive (Table 1). Illness onset ranged from March to December, with 47 (85%) cases having onset during July–September. Thirty-one (56%) patients were male. The median age was 8 years (IQR = 5–80 years), and 51 (93%) were aged <18 years. Fifty-two (95%) patients were hospitalized; none died. Of those hospitalized, 50 (96%) were neuroinvasive disease cases. Incidence of La Crosse virus neuroinvasive disease was highest in Ohio (0.20 per 100,000), West Virginia (0.16), and North Carolina (0.11) (Table 2).

Twenty-three cases of St. Louis encephalitis virus disease were reported, all from Arizona. The median age of patients was 65 years (IQR = 51–73), and 15 (65%) were male. Illness onset date ranged from March to December, with 19 (83%) patients having onset during July–September. Nineteen (83%) cases were neuroinvasive (Table 1). All neuroinvasive disease patients were hospitalized, and none of the nonneuroinvasive disease patients were hospitalized. There were two deaths, in patients aged 67 and 73 years.

Eleven Jamestown Canyon virus disease cases were reported from seven states (Iowa, Massachusetts, Minnesota, New Jersey, Ohio, Wisconsin, and Wyoming). Illness onset ranged from March to December; five cases had onset during April–June and five during July–September. The median age was 56 years (IQR = 41–62 years). Six cases were neuroinvasive, nine patients were hospitalized, and none died.

Seven Powassan virus disease cases were reported from five states (Maine, Massachusetts, New Jersey, New York, and Wisconsin). The median age of patients was 64 years (IQR = 62–75 years), and five patients were male. Six cases were neuroinvasive. All patients were hospitalized, and one died. Illness onset ranged from March to December.

Six cases of eastern equine encephalitis virus disease were reported from four states (Louisiana, Maine, New York, and North Carolina); all were neuroinvasive disease. The median age of patients was 59 years (IQR = 50–78 years), and all patients were male. Illness onset ranged from March to September. All patients were hospitalized, and four died.

In addition to the La Crosse and Jamestown Canyon virus cases, there were four other cases of California serogroup virus disease for which the specific infecting virus was unknown. One case of Cache Valley virus disease was reported from Missouri.

## Discussion

In 2015, WNV remained the most common cause of neuroinvasive arboviral disease in the continental United States and was responsible for 94% of the reported neuroinvasive disease cases. The WNV neuroinvasive disease incidence in 2015 was similar to the median incidence during 2002‒2014 (0.41 per 100,000 population; range = 0.13–1.02) (*3*,*4*). Although the overall case fatality rate for WNV was slightly elevated in 2015 (7%) compared with rates reported previously (median = 5%; range = 3%–15%), the proportion of total cases reported that were neuroinvasive disease also increased, which could account for the higher case fatality rate (*4*). As previously reported, La Crosse virus was the most common cause of neuroinvasive arboviral disease among children (*5*). Four states (Iowa, New Jersey, Ohio, and Wyoming) reported Jamestown Canyon virus for the first time. This likely represents better detection following the routine implementation of Jamestown Canyon virus immunoglobulin M antibody testing at CDC (*6*). All cases of St. Louis encephalitis were reported from Arizona, which experienced a concurrent outbreak of WNV and St. Louis encephalitis virus disease (*7*). Eastern equine encephalitis virus, although rare, remained the most severe domestic arboviral disease, with four deaths reported among six patients.

Arboviruses continue to cause substantial morbidity in the United States, although the reported number of cases varies annually. Cases occur sporadically, and the epidemiology varies by virus and geographic area. Approximately 85% of arboviral disease cases occurred during April–September. Weather, zoonotic host and vector abundance, and human behavior are all factors that can influence when and where outbreaks occur. These factors make it difficult to predict future locations and timing of cases and highlight the importance of surveillance to identify outbreaks and inform public health prevention.

The findings in this report are subject to at least two limitations. First, ArboNET is a passive surveillance system, which leads to an underestimation of the true incidence of disease. To be reported as a disease case, persons must seek care, a clinician must request appropriate diagnostic tests, and health care providers and laboratories need to report cases to public health authorities. Previous studies have estimated that there are 30‒70 nonneuroinvasive disease cases for every reported case of WNV neuroinvasive disease (*8*–*10*). Based on the number of neuroinvasive disease cases reported in 2015, it was expected that 43,650–101,850 nonneuroinvasive disease cases would have occurred; however, only 720 (0.1‒1%) were reported. Second, because ArboNET does not require information about clinical signs and symptoms or laboratory findings, cases might be misclassified.

Health care providers should consider arboviral infections in the differential diagnosis of cases of aseptic meningitis and encephalitis, obtain appropriate specimens for laboratory testing, and promptly report cases to public health authorities (*2*). Because human vaccines against domestic arboviruses are not available, prevention depends on community and household efforts to reduce vector populations (e.g., applying insecticides and reducing breeding sites), personal protective measures to decrease exposure to mosquitoes and ticks (e.g., use of repellents and wearing protective clothing), and screening of blood donors.

**Summary**

**What is already known about this topic?**

Arboviral disease can cause substantial morbidity and mortality in the United States. West Nile virus (WNV) is the leading cause of domestically acquired arboviral disease, but several other arboviruses cause sporadic cases and outbreaks of neuroinvasive disease.

**What is added by this report?**

In 2015, WNV remained the most common cause of neuroinvasive arboviral disease in the United States, with a similar incidence to the median incidence during 2002–2014. In addition, Arizona experienced an outbreak of St. Louis encephalitis virus, and four new states reported their first Jamestown Canyon virus disease cases in 2015.

**What are the implications for public health practice?**

Arboviral diseases are a continuing source of severe illness in the United States each year. Surveillance remains important to identify outbreaks and guide prevention strategies.
